# Real-world lipid outcomes after switching PCSK9-targeting therapies in heterozygous familial hypercholesterolemia: the SHIFT-FH study

**DOI:** 10.1007/s11739-026-04417-8

**Published:** 2026-06-13

**Authors:** Ilenia Lorenza Calcaterra, Carmine De Luca, Luigi Junior Valletta, Guido D’Errico, Martina Ferrandino, Giovanna Cardiero, Maria Donata Di Taranto, Giuliana Fortunato, Gabriella Iannuzzo, Antonella Tufano, Matteo Di Minno

**Affiliations:** 1 Department of Clinical Medicine and Surgery, University of Naples Federico II, Via S. Pansini 5, 80131 Naples, Italy; 2 Regional Reference Centre for Treatment of Dyslipidaemia, Naples, Italy; 3 Department of Molecular Medicine and Medical Biotechnologies, University of Naples Federico II, CEINGE-Biotecnologie Avanzate Franco Salvatore, Naples, Italy

**Keywords:** Familial hypercholesterolemia, PCSK9 inhibition, Inclisiran, Monoclonal antibodies, LDL-C target achievement, Real-world study

## Abstract

Proprotein convertase subtilisin/kexin type 9 (PCSK9) inhibition represents a cornerstone of lipid-lowering therapy in heterozygous familial hypercholesterolemia (HeFH). Monoclonal antibodies (mAbs) and small interfering RNA (siRNA) targeting PCSK9 provide complementary therapeutic strategies; however, real-world data comparing lipid outcomes and target achievement after switching between these treatments remain limited. The SHIFT-FH study was a retrospective real-world cohort study including genetically confirmed HeFH patients receiving maximally tolerated lipid-lowering therapy and stable PCSK9 monoclonal antibody treatment for ≥ 6 months. According to routine clinical practice, patients either switched to siRNA therapy (inclisiran; Group 1) or continued monoclonal antibody therapy (Group 2). The primary outcome was achievement of guideline-recommended LDL-C targets at 6 and 12 months. Secondary outcomes included changes in lipid parameters and hepatic safety. Forty-eight patients were included (22 in Group 1 and 26 in Group 2; mean age 59.3 ± 12.8 years). At baseline, LDL-C target achievement was lower in patients subsequently switched to siRNA therapy (36.4% vs 80.8%; *p* = 0.002). At 12 months, target attainment remained lower in Group 1 (22.7% vs 53.8%). Switching to siRNA therapy was associated with a moderate increase in LDL-C levels (Δ + 29.6 ± 37.4 mg/dL; *p* = 0.003), whereas lipid levels remained stable in patients continuing monoclonal antibody therapy. Exploratory analysis suggested a trend toward an inverse association between baseline LDL-C levels and target achievement at follow-up. Liver enzymes remained within the normal range in both groups. In this real-world cohort of genetically confirmed HeFH patients, LDL-C target achievement was strongly influenced by baseline lipid burden and differed across therapeutic pathways. Switching between PCSK9-targeting strategies may influence long-term lipid control, highlighting the importance of individualized, target-oriented lipid management.

**Supplementary Information:**

The online version contains supplementary material available at https://doi.org/10.1007/s11739-026-04417-8.

## Introduction

Heterozygous familial hypercholesterolemia (HeFH) is a genetic disorder characterized by lifelong elevation of low-density lipoprotein cholesterol (LDL-C), leading to a markedly increased risk of premature atherosclerotic cardiovascular disease (ASCVD) [1] with a prevalence of 1 in 220 individuals worldwide, recognized as the most common hereditary lipid disorder [2], that often remains underdiagnosed and undertreated [3]. Despite maximally standard lipid-lowering therapy (LLT) with statins and ezetimibe, a significant percentage of HeFH patients fail to achieve recommended LDL-C goal [4]. The 2025 ESC/EAS Guidelines for the management of dyslipidemias highlight the need for early and intensive LDL-C lowering in HeFH classified as high-risk patients, advocating the stepwise combination of high-intensity statins, ezetimibe, and PCSK9 inhibitors [5]. Two clinically validated pharmacologic strategies targeting PCSK9 are currently available: monoclonal antibodies (mAb), which neutralize circulating PCSK9, and small interfering RNA (siRNA), which suppress hepatic PCSK9 synthesis [6]. Both approaches have demonstrated substantial LDL-C reductions and cardiovascular benefit in randomized trials [7–12]; however, real-world comparative evidence remain limited. In clinical practice, treatment selection relies on both patient preferences and clinician judgment, the latter being primarily informed by the patient’s adherence, overall compliance, and quality of life, with the additional aim of minimizing dosing frequency whenever appropriate.

Early observational studies have explored inclisiran use in mixed cardiovascular populations, reporting consistent LDL-C reductions but variable results when compared indirectly with mAb therapy [13, 14]. Nevertheless, these investigations did not specifically target individuals with HeFH and offered only a limited appraisal of hepatic safety. In addition, the preponderance of available evidence stems from short-duration studies or methodologically heterogeneous cohorts, without addressing treatment persistence or switching behaviors within real-world clinical practice settings.

To the best of our knowledge, no prior investigation has specifically described lipid outcomes and hepatic safety in real-world HeFH patients undergoing to different PCSK9 inhibition strategies, including treatment switching. Accordingly, the present study was aimed to provide pragmatic real-world data on lipid profile evolution and hepatic safety in HeFH patients managed with different PCSK9 inhibition strategies in routine clinical practice.

## Methods

The SHIFT-FH study (SwItcHing between pcSk9-targeting Therapies in Familial Hypercholesterolemia) was designed as a retrospective real-world cohort study, conducted and reported in accordance with the STROBE (Strengthening the Reporting of Observational Studies in Epidemiology) checklist [15]. Between 2023 and 2025, within the framework of the LIPIGEN network, a national project on familial dyslipidemia [16], medical records of patients attending the lipid clinic of Federico II University Hospital were retrospectively reviewed to identify individuals with very high levels of LDL-C (above the 95th percentile compared with a sex- and age-matched general population), normal triglyceride (TG) levels, and presumed autosomal dominant transmission of hypercholesterolemia (Dutch Lipid Clinic Network score > 6).

### Study population

The major inclusion criteria were: genetically confirmed diagnosis of HeFH (presence of a single pathogenic variant in FH-causative genes) and stable treatment with a PCSK9 monoclonal antibody (alirocumab or evolocumab) for at least 6 months prior to data review, on top of maximum tolerated LLT. Genetic confirmation of HeFH was obtained through molecular testing performed by analyzing the main genes associated with familial hypercholesterolemia (*LDLR*, *APOB*, *PCS*K9, LDLRAP1, and *APOE*) by next-generation sequencing using the Devyser FH v2 kit (Devyser, Sweden) for target enrichment as previously described [16, 17]. Exclusion criteria included: age < 18 years; inability to understand or provide informed consent; moderate hypertriglyceridemia (TG > 300 mg/dL); active hepatic or inflammatory disease [defined as acute or chronic hepatitis associated with transaminase levels > 3 × the upper limit of normal, (ULN)]; end-stage kidney disease (estimated glomerular filtration rate < 15 mL/min/m^2^, due to the lack of evidence in patient with extremely reduced estimated glomerular filtration rate) according to the most recent guidelines [18]; secondary causes of hypercholesterolemia (e.g., hypothyroidism, hormone therapy, corticosteroid use); diabetes mellitus with HbA1c > 7.0% [19], discontinuation of LLT during the observation period; incomplete follow-up data; and clinical or familial evidence of familial combined hyperlipidemia. Due to the retrospective nature of the study and the real-world setting, the sample size was not predetermined; all consecutive eligible patients attending the lipid clinic during the study period were included.

### Study protocol

Following the acquisition of informed consent, comprehensive medical history was obtained from existing clinical records for each patient. Retrospective data encompassed demographic variables (age, sex), vascular risk factors (smoking status, hypertension, diabetes mellitus), concomitant clinical conditions, body mass index (BMI), and both last follow-up and prior LLT data.

Biochemical data on lipid parameters [total cholesterol (TC); LDL-C; high-density lipoprotein cholesterol (HDL-c); TG] and hepatic necrosis markers [aspartate aminotransferase (AST) and alanine aminotransferase (ALT)] were retrospectively retrieved at baseline (T0), 6 months (T1), and 12 months (T2), when available. Baseline (T0) was defined as the visit occurring 6 months before T1 and 12 months before T2, ensuring temporal comparability between groups, considering that routinary follow-up in our Lipid Clinic Center is scheduled every 6 months. Analyses at each follow‑up timepoint were conducted exclusively on the available paired measurements; consequently, the number of patients included in the evaluations differed across timepoints.

Given the retrospective nature of the study, patients were classified according to the therapeutic pathway followed in routine clinical practice. One group switched to PCSK9-targeting siRNA therapy (inclisiran 284 mg, following the standard dosing schedule at day 1, month 3, and every 6 months thereafter), whereas the other continued treatment with PCSK9 monoclonal antibodies (alirocumab 150 mg or evolocumab 140 mg was administered every 14 days according to approved dosing schedules). Following eligibility screening, enrolled patients were classified into two groups according to the therapeutic pathway observed in routine clinical practice. Group 1 (G1) included patients who switched from PCSK9 monoclonal antibody therapy (alirocumab or evolocumab) to small interfering RNA therapy (inclisiran), while Group 2 (G2) included patients who continued PCSK9 monoclonal antibody treatment. Baseline (T0) was defined as the last available clinical and biochemical assessment preceding treatment switching for G1, or the corresponding timepoint during ongoing PCSK9 monoclonal antibody therapy for G2. Treatment allocation was not randomized. The decision to switch was based on clinician judgment and patient preference, primarily aimed at reducing treatment burden and improving long-term adherence. Accordingly, differences in baseline lipid control between groups were expected and were considered in the interpretation of follow-up findings.

### Outcomes and statistical analysis

The primary outcome was the proportion of patients achieving LDL-C targets according to the 2025 ESC/EAS Guidelines at 6 and 12 months (T1 and T2) [5]. In accordance with guideline recommendations, LDL-C targets were defined as < 55 mg/dL for very high-risk patients and < 70 mg/dL for high-risk patients [5]. Patients were classified according to their individual cardiovascular risk profile as defined by the ESC/EAS guidelines [5]. Secondary outcomes included changes in lipid parameters (LDL-C, HDL-C, TC, TG) and hepatic safety based on variations in AST and ALT. All data were retrospectively extracted from electronic medical and laboratory records. Continuous variables were summarized as mean ± standard deviation (SD) or median (interquartile range, IQR), and categorical variables as counts and percentages. Normality was assessed using the Shapiro–Wilk test. Between-group comparisons were performed with Student’s t test or Mann–Whitney U test, as proper. Within-group comparisons used paired t test or Wilcoxon signed-rank test. Categorical variables were analyzed using Chi-square or Fisher’s exact test. A two-tailed *p* value < 0.05 was considered statistically significant. No imputation of missing data was performed. Given the retrospective real-world design, analyses at each follow-up timepoint were conducted using a complete-case approach including only patients with available paired biochemical measurements. Comparisons of target achievement between groups were conducted using tests for independent proportions, as treatment allocation defined the groups rather than paired observations. Changes in target status over follow‑up were assessed by examining the proportion of patients transitioning between categories and illustrated through a Sankey diagram representing these variations. Due to the small sample size, the retrospective design, and the clinically driven treatment allocation, no propensity score-based adjustment was applied, as it was deemed inappropriate for the study objectives. An exploratory univariate logistic regression analysis was performed to assess the association between baseline LDL-C levels and LDL-C target achievement at T2 for G1. All statistical analyses and graphical representations were performed using R software (R Foundation for Statistical Computing, Vienna, Austria; version 4.5.2) within the RStudio environment.

## Results

A total of 228 patients were screened for eligibility for inclusion in the SHIFT-FH study cohort. Of these, patients were excluded according to predefined criteria: 73 for Dutch Lipid Clinic Network score < 6, 18 for diabetes mellitus with HbA1C > 7%, 48 for combined hyperlipidemia with hypertriglyceridemia, 29 for secondary hypercholesterolemia, and 12 for a history of malignant neoplasia within the previous two years. Forty-eight patients met the inclusion criteria and constituted the final SHIFT-FH study cohort (Supplementary Fig. 1). Enrolled patients were classified into two groups: G1 included 22 patients who switched from PCSK9 monoclonal antibody therapy to siRNA (inclisiran), and G2 included 26 patients who continued PCSK9 monoclonal antibody therapy. All patients were in combined treatment with Ezetimibe 10 mg and statin. Of them, 72.9% on high-intensity statin treatment, while 27.1% on treatment with moderate intensity statin. No patient on treatment with Bempedoic Acid (Table 1).

**Table 1:** Baseline clinical and biochemical characteristics of the study population

	Total (48)	Group 1 (22)	Group 2 (26)	*p* value
Age (*n*)	59.3 ± 12.8	58.9 ± 13	61.4 ± 12.5	0.228
Male (%, *n*)	54.2% (26)	54.5% (12)	53.8% (14)	0.961
BMI (Kg/m^2^)	26.0 ± 1.9	26.0 ± 2.1	26.1 ± 1.8	0.829
Smoking habits (%, *n*)	45.8% (22)	36.4% (8)	53.8% (14)	0.226
Arterial hypertension (%, *n*)	50.0% (24)	40.9% (9)	57.7% (15)	0.247
*LDLR* causative variants (%,*n*)^a^	100% (48)	100% (22)	100% (26)	N.A
DUTCH score	15.0 ± 4.0	16 ± 3.7	14.0 ± 4.1	0.86
Diabetes mellitus (%, *n*)	10.4% (5)	9.1% (2)	11.5% (3)	0.782
Secondary prevention (%, *n*)	50.0% (24)	45.5% (10)	53.8% (14)	0.562
Total cholesterol (mg/dl)	139.2 ± 43.5	150.9 ± 47.5	129.3 ± 37.9	0.088
Triglycerides (mg/dl)^b^	87.5 [61.7–107.7]	83 [61.5–108]	95.5 [63.5–106]	0.992
HDL-C (mg/dl)	55.6 ± 13.3	55.1 ± 14.8	56.0 ± 12.2	0.824
LDL-C (mg/dl)	67.0 ± 43.8	76.2 + 42	59.2 ± 44.6	0.184
AST (U/dl)^a^	24.0 [22.7;25.0]	22.5 [20.2;23.7]	25 [23.2;25.7]	0.013
ALT (U/dl)^a^	19.0 [18.0;20.0]	20.0 [18.2;20]	19.0 [18.0;20.0]	0.432
Therapeutic LDL-C target (%, *n*)	60.4% (29)	36.4% (8)	80.8% (21)	0.002
Ezetimibe 10 mg (%)	100% (48)	100% (48)	100% (48)	N.A
High-intensity statin (%,*n*)^c^	72,9% (35)	81.8% (18)	65.4% (17)	0.202
Moderate-intensity statin (%,*n*)^d^	27.1% (13)	18.2% (4)	18.2% (9)	0.202

^a^see Suppl.Tab.1 for distribution of mutation patterns. ^b^reported as median [IQR]. ^c^High-intensity statin: Atorvastatin 40 mg or Rosuvastatin 20 mg. ^d^Moderate-intensity statin: Atorvastatin 20 mg or Rosuvastatin 10 mg

Continuous variables are expressed as mean ± standard deviation or median [interquartile range], as appropriate. Categorical variables are reported as percentages and absolute numbers. Comparisons between groups were performed using Student’s *t* test or Mann–Whitney *U* test for continuous variables, and χ^2^ test for categorical variables. *BMI* body mass index, *ALT* alanine aminotransferase, *AST* aspartate aminotransferase, *HDL-C* high-density lipoprotein cholesterol, *LDL-C* low-density lipoprotein cholesterol, *N.A.* not applicable

### Clinical and biochemical profile at baseline

Baseline clinical and biochemical characteristics of the SHIFT-FH study cohort are reported in Table 1. All patients included in the study were heterozygous carriers of pathogenic *LDLR* variants (100%, 48) (Table 1). All patients carried pathogenic variants in the LDLR gene. Missense variants were observed in 15 patients (68.2%) in Group 1 and in 18 patients (69.2%) in Group 2, while null variants were present in 7 (31.8%) and 8 (30.8%) patients, respectively. The distribution of mutation types did not differ between treatment groups **(**Supplementary Table 1**).** The proportion of patients achieving the therapeutic LDL-C target was significantly lower in G1 than in G2 (36.4% vs. 80.8%, *p* = 0.002), indicating a marked imbalance in baseline lipid control between the two groups. No significant differences were observed between groups with respect to age, sex, cardiovascular risk factors (arterial hypertension, smoking habits, diabetes mellitus), DUTCH score, TC, HDL-C, TG and LDL-C. In contrast, AST levels were significantly higher in Group 2 compared with Group 1 [22.5 (20.2;23.7) vs 25 (23.2;25.7) U/L, *p* = 0.013), although values remained within the normal range in both groups.

### Clinical and biochemical profile at T1

**Fig. 1:**
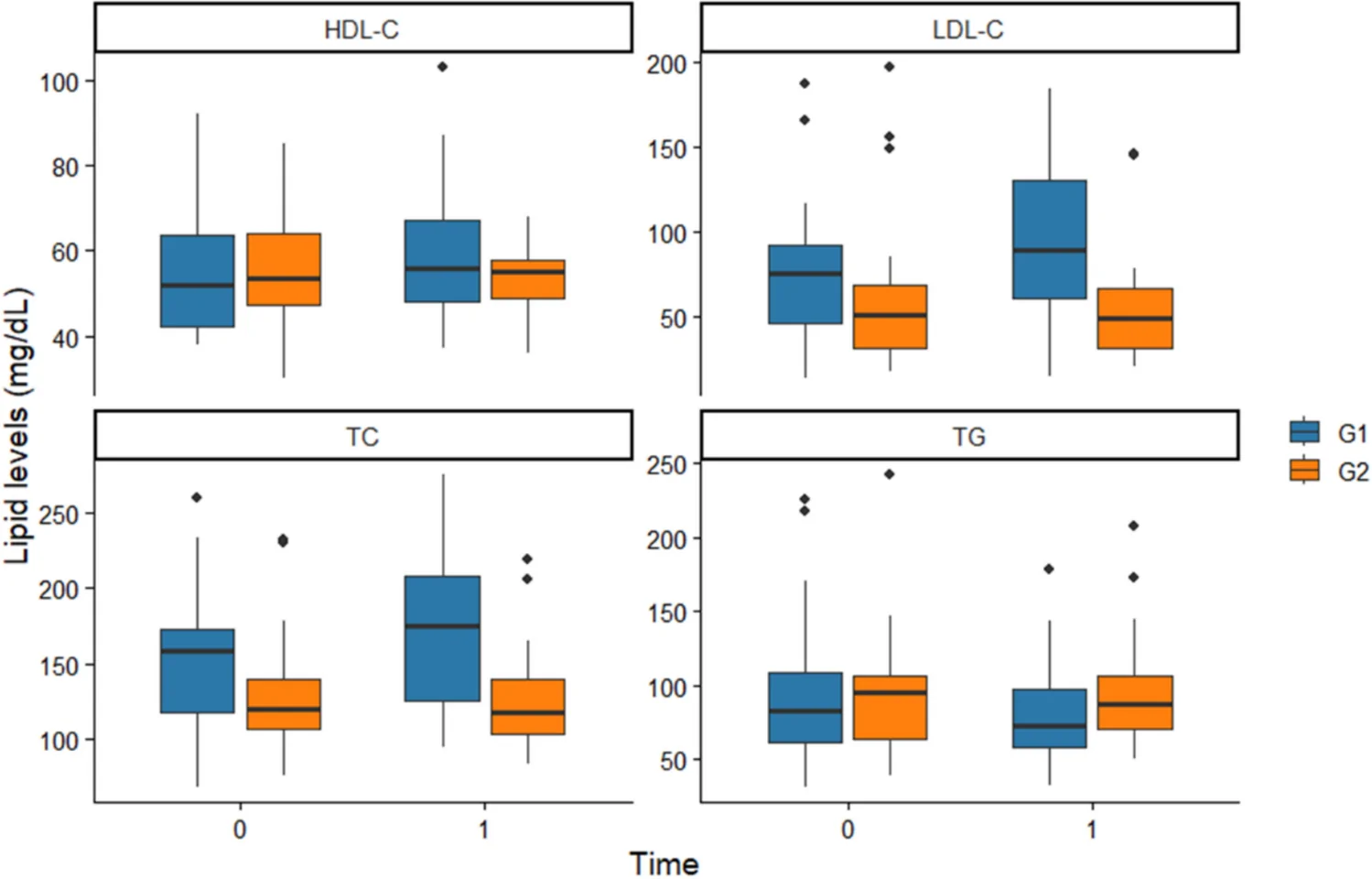
Distribution of lipid parameters at baseline (T0) and follow-up (T1) according to study group. Boxplots illustrate the distribution of total cholesterol (TC), triglycerides (TG), HDL-C, and LDL-C at baseline (T0) and first follow-up (T1) in the two study groups (G1 and G2). Boxes represent the interquartile range (IQR) with the median indicated by the horizontal line; whiskers extend to 1.5 × IQR and points represent outliers. Lipid concentrations are expressed in mg/dL

The number of patients with available paired biochemical measurements remained stable across follow‑up, except for a single missing laboratory assessment in G1. At T1, 84.6% of patients in G2 and 47.6% of those in G1 achieved LDL-C targets, with no significant changes compared with baseline (*p* = 0.414 for G1 and *p* = 0.564 for G2). No statistically significant within-group variations in lipid parameters or liver enzymes were observed in either cohort at this timepoint (Table 2). In G1, mean TC at T1 was 173.2 ± 55.1 mg/dL, median TG were 73 [58–97] mg/dL, HDL-C was 60.2 ± 17.9 mg/dL, and LDL-C was 95.2 ± 48.4 mg/dL, with median AST and ALT levels of 23 [21–24] U/L and 20 [19–21] U/L, respectively. In G2, mean TC was 125.9 ± 33.5 mg/dL, TG 87 [70–106.5] mg/dL, HDL-C 53.4 ± 7.8 mg/dL, and LDL-C 53.6 ± 31.8 mg/dL (Fig. 1); AST and ALT values were 23.5 [23–25] U/L and 19 [19, 20] U/L, respectively. A modest, non-significant increase in TC and LDL-C was observed in G1, whereas lipid parameters in G2 remained stable throughout follow-up (Table 2).

**Table 2:** Six-month follow-up lipid profile and liver enzyme levels according to treatment group

	G1 (21)		G2 (26)	
	Δ vs baseline	*p* value	Δ vs baseline	*p* value
CT (mg/dl)	21.1 ± 48.0	0.057	− 3.4 ± 38.5	0.654
TG (mg/dl)	− 12.8 ± 44.7	0.204	0.3 ± 48.8	0.971
HDL-C (mg/dl)	5.1 ± 14.9	0.129	− 2.6 ± 12.1	0.286
LDL-C (mg/dl)	18.1 ± 42.6	0.065	− 5.6 ± 42.2	0.504
AST (U/dl)	1.1 ± 5.0	0.334	− 1.2 ± 4.1	0.159
ALT (U/dl)	1.3 ± 4.2	0.195	− 0.3 ± 2.2	0.424
LDL-C target (%, *n*)	47.6% (10)	0.414	84.6% (22)	0.564

Changes from baseline (Δ) and corresponding two-sided *p* values for within-group comparisons are reported for both groups. Continuous variables are expressed as mean ± standard deviation. Within-group comparisons were performed using paired tests, as appropriate. *ALT* alanine aminotransferase, *AST* aspartate aminotransferase, *CT* total cholesterol, *HDL-C* high-density lipoprotein cholesterol, *LDL-C* low-density lipoprotein cholesterol, *TG* triglycerides

### Clinical and biochemical profile at T2

**Fig. 2:**
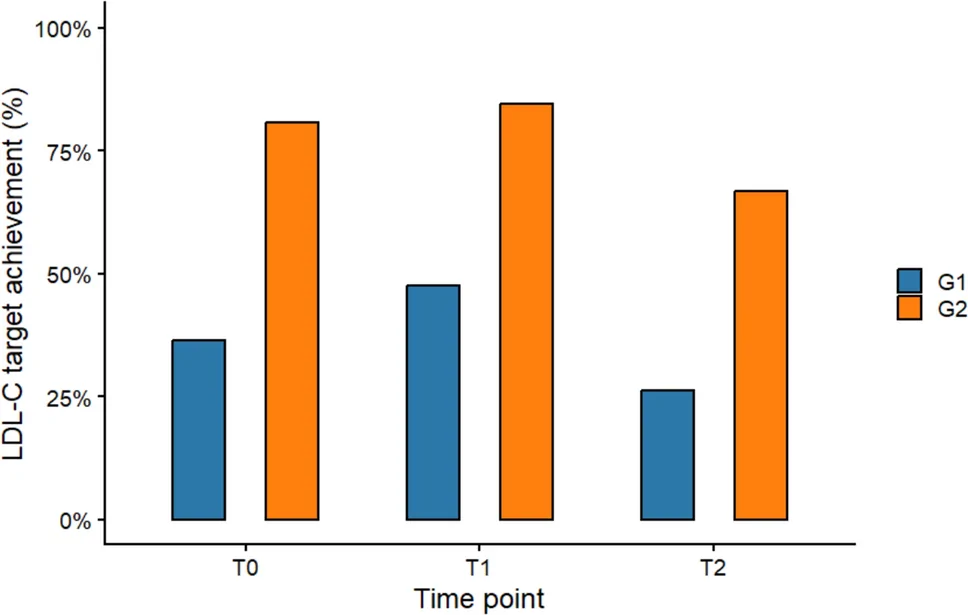
LDL-C target achievement over time in the two study groups. Proportion of patients achieving the therapeutic LDL-C target at baseline (T0), 6 months (T1), and 12 months (T2) in G1 and G2. P values refer to between-group comparisons at each time point (independent proportions test)

At the 12 month follow-up (T2), paired biochemical measurements were available for 19 patients in G1 and 21 patients in G2. The proportion of patients achieving LDL‑C targets remained unchanged over time, with 26.3% in G1 and 73.7% in G2 (Fig. 2). In G1, mean TC was 181.3 ± 47.8 mg/dL, TG were 73 [61.5–107]  mg/dL, HDL‑C was 60.0 ± 19.7 mg/dL, and LDL‑C was 103.5 ± 36.1 mg/dL. Median AST and ALT levels were 22 [24]  U/L and 19 [18–20] U/L, respectively. In G2, mean TC was 134.6 ± 32.0 mg/dL, median TG were 103 [68.8–145.3] mg/dL, HDL‑C was 50.4 ± 10.6 mg/dL, and LDL‑C was 62.1 ± 27.0 mg/dL, with corresponding median AST and ALT levels of 22 [19.5–23.5] U/L and 19 [19, 20] U/L. In G1, a significant increase in TC and LDL‑C was observed compared with baseline (Δ = 31.2 ± 41.9 mg/dL, *p* = 0.004; and Δ = 29.6 ± 37.4 mg/dL, *p* = 0.003), whereas no significant changes were detected in TG, HDL‑C, or liver enzyme levels. At 12 months of follow‑up, divergent trends were observed between the two treatment groups, while a statistically significant reduction in AST values was noted, all measurements consistently remained within the normal range throughout the study period (Table 3). The individual LDL-C trajectories across the study time points are illustrated in Supplementary Fig. 2, highlighting the variability of lipid responses within each treatment group.

**Table 3:** Twelve-month follow-up outcomes and changes from baseline according to treatment group

	G1 (19)		G2 (21)	
	Δ vs baseline	*p* value	Δ vs baseline	*p* value
CT (mg/dl)	31.2 ± 41.9	0.004	1.4 ± 44.2	0.894
TG (mg/dl)	0.3 ± 39.8	0.973	18.9 ± 43.5	0.074
HDL-C (mg/dl)	4.5 ± 15.6	0.227	−6.9 ± 14.1	0.46
LDL-C (mg/dl)	29.6 ± 37.4	0.003	−2.4 ± 50	0.839
AST (U/dl)	0.21 ± 3.5	0.796	−3.21 ± 3.3	< 0.001
ALT (U/dl)	−0.21 ± 2.5	0.719	0.6 ± 2.2	0.22
LDL-C Target (%, *n*)	26.3% (5)	0.257	73.7% (14)	1.00

Changes from baseline (Δ) and corresponding two-sided *p* values are reported for Group 1 and Group 2. Continuous variables are expressed as mean ± standard deviation. Within-group comparisons were performed using paired tests, as appropriate. *ALT* alanine aminotransferase, *AST* aspartate aminotransferase, *CT* total cholesterol, *HDL-C* high-density lipoprotein cholesterol, LDL-C low-density lipoprotein cholesterol, *TG* triglycerides

### Changes in target status during follow-up

**Fig. 3:**
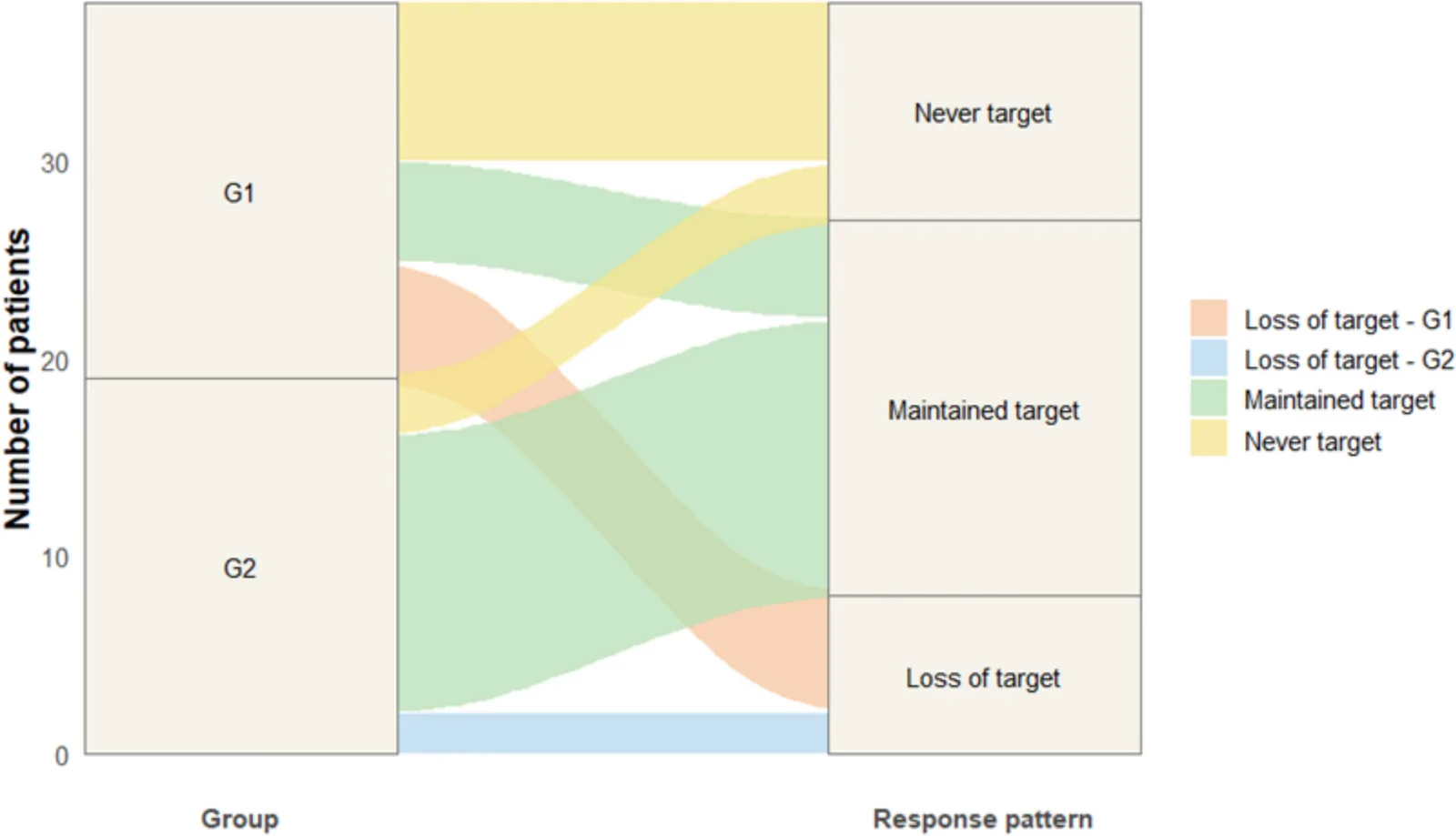
Sankey diagram showing patterns of therapeutic target achievement according to treatment group. Patients from each treatment group (G1 and G2) were classified according to target attainment over time (T0, T1, and T2) into three response patterns: never target (no target attainment at any time point), maintained target (target achieved at T1 and maintained at T2), and loss of target (transition from target attainment to loss of control during follow-up). The width of each flow is proportional to the number of patients in each trajectory

Among patients with available data, 47.4% (18/38) were classified as never target, while 14.6% (6/41) experienced a loss of target during follow-up. The proportion of never target patients differed significantly between treatment groups (difference 42.1%, *p* = 0.009), whereas the difference in loss of target was not statistically significant (difference 17.5%, *p* = 0.115) (Fig. 3).

### Exploratory analysis of predictors of LDL-C response

In the exploratory univariate logistic regression analysis, higher baseline LDL-C levels showed a trend toward a lower likelihood of achieving LDL-C targets at T2 (OR = 0.96 per 1 mg/dL increase; 95% CI 0.91–1.00; *p* = 0.068).

## Discussion

In the SHIFT-FH study cohort, we assessed LDL-C target achievement, lipid profile evolution, and hepatic safety over 12 months in genetically confirmed HeFH patients treated with two PCSK9 inhibition strategies (siRNA vs mabs).

LDL-C target achievement differed substantially between groups already at baseline, with a markedly lower proportion of patients at goal in those subsequently switched to inclisiran, with a higher proportion of patients on continued mAbs therapy meeting guideline-recommended targets compared with those later switched to siRNA, and this imbalance persisted over follow-up. This pattern is consistent with the well-described gap between recommended LDL-C goals and real-world attainment in high/very-high-risk populations, even in contemporary European settings [20].

The marked imbalance in LDL-C target achievement observed at baseline deserves careful consideration when interpreting between-group differences. Because treatment allocation was clinician-driven rather than randomized, patients switched to inclisiran had poorer LDL-C control at baseline, suggesting the presence of confounding by indication, and may have represented a subgroup with less satisfactory lipid control, greater residual LDL-C burden, or specific management needs in routine practice. Consequently, the two groups cannot be considered fully comparable, and the observed differences in lipid outcomes may reflect baseline disease severity rather than the treatment strategy itself. This potential selection bias likely contributed, at least in part, to the different lipid trajectories observed and results should be interpreted cautiously and not be considered evidence of a causal effect of treatment switching, however in the frame of real-world practice.

Exploratory regression analysis suggested a trend toward an inverse relationship between baseline LDL-C levels and the likelihood of achieving LDL-C targets at 12 months. This observation aligns with recent real-world evidence identifying baseline LDL-C as a major determinant of target non-achievement in PCSK9 inhibitor treatment pathways [21]. Clinically, this relationship is intuitive within a target-based framework: markedly elevated baseline LDL-C requires a larger absolute reduction to meet stringent guideline thresholds, making residual above-target values more likely even when relative LDL-C lowering is substantial. Furthermore, evidence suggests that baseline LDL-C influences the percentage reduction observed across multiple lipid-lowering drug classes, including PCSK9 inhibitors, reinforcing its role as a key driver of treatment response and goal attainment [22].

When examining lipid profile trajectories, patients who continued monoclonal antibody therapy maintained stable LDL-C levels throughout follow-up, whereas those who switched to siRNA therapy showed a progressive increase in LDL-C and TC at 12 months. Although the LDL-C increase observed after switching reached statistical significance, its magnitude remained relatively modest and should be interpreted within the context of baseline lipid burden and pharmacological differences between treatment strategies. This pattern aligns with real-world evidence on inclisiran, which while generally supporting meaningful LDL-C reductions also reports variability related to background LLT and, notably, prior treatment with PCSK9 mAbs [23]. Consistently, other observational cohorts have documented that patients transitioning from mAbs to siRNA may show an LDL-C increase, mirroring the trend observed in our study [13].

A plausible explanation for these divergent lipid trajectories lies in the distinct pharmacodynamic mechanisms of the two therapeutic strategies. MAbs (evolocumab and alirocumab) act extracellularly by binding circulating PCSK9, thereby preventing PCSK9-mediated degradation of the LDL receptor (LDLR) and enhancing hepatic LDLR recycling and LDL clearance. Because they directly neutralize circulating PCSK9, their onset of action is rapid and their lipid-lowering effect reflects sustained antibody availability in the circulation [24]. In contrast, inclisiran is a GalNAc-conjugated siRNA selectively internalized by hepatocytes, where it silences PCSK9 synthesis via RNA interference (RISC-mediated mRNA degradation). This mechanism reduces hepatic PCSK9 production and progressively decreases circulating PCSK9 levels over time, without binding or inactivating existing extracellular PCSK9 molecules [25]. Conceptually, this “inside-the-hepatocyte” approach depends on intracellular delivery and sustained gene silencing rather than immediate extracellular neutralization. As a result, switching from mAbs to siRNA represents a pharmacodynamic shift that, in real‑world transitions without therapeutic overlap, may transiently alter circulating PCSK9 dynamics and contribute to LDL‑C variations observed during follow‑up.

Across randomized clinical trials, mAbs generally achieve slightly greater mean LDL-C reductions than inclisiran (approximately 55–60% vs. 45–50%), although both strategies provide clinically meaningful lipid lowering [7–12], suggesting that a relative decrease in pharmacologic potency may become clinically evident when switching from mAb to siRNA therapy. This effect is particularly relevant in patients who are already near guideline-recommended thresholds or those with higher baseline LDL-C values, who require larger absolute reductions to remain on target [24]. Supporting this interpretation, comparative real-world analyses indicate higher rates of LDL-C target attainment with monoclonal antibodies than with inclisiran among patients starting from elevated baseline LDL-C levels [26].

Considering hepatic safety, both strategies showed an excellent tolerability profile. Transaminases remained within the normal range throughout follow-up and no clinically relevant elevations were observed. This is consistent with the broader evidence base indicating a favorable hepatic safety profile for both monoclonal antibody PCSK9 inhibitors and inclisiran in clinical development and real-world settings [24].

The comparison between treatment strategies should be interpreted with caution, as treatment allocation was clinician- and patient-driven rather than randomized. In routine clinical practice, switching from PCSK9 mAbs to siRNA is often prompted by the desire to simplify the therapeutic regimen and potentially improve long-term adherence. Therefore, the LDL-C increase observed after switching should not be interpreted as evidence of reduced drug efficacy, but rather as the combined result of baseline lipid burden, pharmacological differences between treatment strategies, and real-world treatment allocation.

This study has limitations inherent to its retrospective, single‑center design and modest sample size, which restrict causal inference and limit generalizability. Treatment allocation was non‑randomized, adherence could not be objectively quantified, and cardiovascular outcomes were not assessed. The relatively small sample size and the reduction in patient numbers at T2 limit the statistical power of the study and preclude robust multivariable adjustment for T0 differences. Adherence data were not systematically available in this retrospective analysis and, therefore, represent a limitation, particularly considering the reduced injection frequency of siRNA. Notably, the lower proportion of patients achieving LDL-C targets in the switching group should be interpreted considering the markedly lower target attainment already present at baseline. This pattern may reflect a subgroup of patients with more difficult-to-control lipid profiles or reduced responsiveness to PCSK9-targeting therapies. In such contexts, additional lipid-lowering strategies, including emerging third-line treatments such as angiopoietin-like protein 3 (ANGPTL3) inhibitors, may represent a potential therapeutic option [25]. Nevertheless, the focus on a genetically confirmed HeFH cohort and the pragmatic observation of real‑world switching behavior provide clinically relevant insights into target attainment, lipid trajectories, and hepatic tolerability in routine practice. Therefore, the findings should not be considered as hypothesis-generating.

## Conclusions

In conclusion, in the SHIFT-FH study cohort, LDL-C target achievement was strongly influenced by baseline lipid burden and differed across therapeutic pathways. Patients who continued mAbs therapy maintained stable lipid control, whereas those who switched to siRNA therapy showed a moderate increase in LDL-C levels at 12 months, while maintaining an excellent hepatic safety profile. These findings highlight the importance of individualized, target-oriented lipid management when considering therapeutic switching between PCSK9-targeting strategies. Future prospective multicenter studies including cardiovascular outcomes are warranted to better define the optimal positioning of these therapies in routine clinical practice.

## Supplementary Information

Below is the link to the electronic supplementary material.

Supplementary file1 (DOCX 49 KB)

## Data Availability

The data that support the findings of this study are available from the corresponding author upon reasonable request.

## Declarations

### AI-assisted writing

During the preparation of this manuscript, the authors used ChatGPT (OpenAI) to assist in improving the language, structure, and clarity of the text. The AI tool was used exclusively for language polishing and editorial refinement. No scientific content, data analysis, or interpretation was generated by the AI. All outputs were carefully reviewed, edited, and validated by the authors, who take full responsibility for the integrity and accuracy of the final manuscript.

## References

[1] McGowan MP, Hosseini Dehkordi SH, Moriarty PM, Duell PB (2019) Diagnosis and treatment of heterozygous familial hypercholesterolemia. J Am Heart Assoc 8(24):e013225 10.1161/JAHA.119.013225 PMC6951065 31838973

[2] Polychronopoulos G, Tzavelas M, Tziomalos K (2021) Heterozygous familial hypercholesterolemia: prevalence and control rates. Expert Rev Endocrinol Metab 16(4):175–179 10.1080/17446651.2021.1929175 33993819

[3] Sturm AC, Knowles JW, Gidding SS, Ahmad ZS, Ahmed CD, Ballantyne CM et al (2018) Clinical genetic testing for familial hypercholesterolemia: JACC scientific expert panel. J Am Coll Cardiol 72(6):662–680 10.1016/j.jacc.2018.05.044 30071997

[4] Jianu N, Nitu ET, Merlan C, Nour A, Buda S, Suciu M et al (2025) A Comprehensive review of the latest approaches to managing hypercholesterolemia: a comparative analysis of conventional and novel treatments: part II. Pharmaceuticals (Basel) 18(8):1150 10.3390/ph18081150 PMC12389475 40872541

[5] Mach F, Koskinas KC, Roeters van Lennep JE, Tokgozoglu L, Badimon L, Baigent C et al (2025) Focused update of the 2019 ESC/EAS guidelines for the management of dyslipidaemias. Eur Heart J 46(42):4359–4378 10.1093/eurheartj/ehaf190 40878289

[6] Catapano AL, Pirillo A, Norata GD (2020) New pharmacological approaches to target PCSK9. Curr Atheroscler Rep 22(7):24 10.1007/s11883-020-00847-7 32495301

[7] Geba GP, Mohammadi KA, Damask A, Paulding C, Lotta LA, Hindy G et al (2025) Effect of pcsk9 inhibition with alirocumab in patients with probable familial hypercholesterolemia or type iii hyperlipoproteinemia: results from the ODYSSEY OUTCOMES trial. J Am Heart Assoc 14(17):e041190 10.1161/JAHA.124.041190 PMC12553435 40847481

[8] Sabatine MS, Giugliano RP, Keech AC, Honarpour N, Wiviott SD, Murphy SA et al (2017) Evolocumab and clinical outcomes in patients with cardiovascular disease. N Engl J Med 376(18):1713–1722 10.1056/NEJMoa1615664 28304224

[9] Santos RD, Stein EA, Hovingh GK, Blom DJ, Soran H, Watts GF et al (2020) Long-term Evolocumab in patients with Familial Hypercholesterolemia. J Am Coll Cardiol 75(6):565–574 10.1016/j.jacc.2019.12.020 32057369

[10] Sinnaeve PR, Schwartz GG, Wojdyla DM, Alings M, Bhatt DL, Bittner VA et al (2020) Effect of alirocumab on cardiovascular outcomes after acute coronary syndromes according to age: an ODYSSEY OUTCOMES trial analysis. Eur Heart J 41(24):2248–2258 10.1093/eurheartj/ehz809 PMC7308542 31732742

[11] Ray KK, Troquay RPT, Visseren FLJ, Leiter LA, Scott Wright R, Vikarunnessa S et al (2023) Long-term efficacy and safety of inclisiran in patients with high cardiovascular risk and elevated LDL cholesterol (ORION-3): results from the 4 year open-label extension of the ORION-1 trial. Lancet Diabetes Endocrinol 11(2):109–119 10.1016/S2213-8587(22)00353-9 36620965

[12] Ray KK, Landmesser U, Leiter LA, Kallend D, Dufour R, Karakas M et al (2017) Inclisiran in patients at high cardiovascular risk with elevated LDL cholesterol. N Engl J Med 376(15):1430–1440 10.1056/NEJMoa1615758 28306389

[13] Mulder J, Galema-Boers AMH, Roeters van Lennep JE (2023) First clinical experiences with inclisiran in a real-world setting. J Clin Lipidol 17(6):818–827 10.1016/j.jacl.2023.09.005 37775462

[14] Ho VQT, Tran NB, Nguyen N, Arrighini GS, Downes D, Dandamudi M et al (2025) PCSK9 targeting therapies for familial hypercholesterolaemia: a meta-analysis of efficacy on lipid biomarkers and safety in adults and children across 23 RCTs. Open Heart. 12(2):e003490 10.1136/openhrt-2025-003490 PMC12374655 40841123

[15] von Elm E, Altman DG, Egger M, Pocock SJ, Gotzsche PC, Vandenbroucke JP et al (2007) Strengthening the reporting of observational studies in epidemiology (STROBE) statement: guidelines for reporting observational studies. BMJ 335(7624):806–808 10.1136/bmj.39335.541782.AD PMC2034723 17947786

[16] Pirillo A, Garlaschelli K, Arca M, Averna M, Bertolini S, Calandra S et al (2017) Spectrum of mutations in Italian patients with familial hypercholesterolemia: new results from the LIPIGEN study. Atheroscler Suppl 29:17–24 10.1016/j.atherosclerosissup.2017.07.002 28965616

[17] Cardiero G, Ferrandino M, Calcaterra IL, Iannuzzo G, Di Minno MND, Buganza R et al (2024) Impact of 12-SNP and 6-SNP polygenic scores on predisposition to high LDL-cholesterol levels in patients with familial hypercholesterolemia. Genes. 10.3390/genes15040462 PMC11050365 38674396

[18] Kidney Disease: Improving Global Outcomes CKDWG (2024) KDIGO 2024 clinical practice guideline for the evaluation and management of chronic kidney disease. Kidney Int 105(4S):S117–S314 10.1016/j.kint.2023.10.018 38490803

[19] Davies MJ, Aroda VR, Collins BS, Gabbay RA, Green J, Maruthur NM et al (2022) Management of hyperglycaemia in type 2 diabetes a consensus report by the american diabetes association (ADA) and the European association for the study of diabetes (EASD). Diabetologia 65(12):1925–1966 10.1007/s00125-022-05787-2 PMC9510507 36151309

[20] Ray KK, Haq I, Bilitou A, Manu MC, Burden A, Aguiar C et al (2023) Treatment gaps in the implementation of LDL cholesterol control among high- and very high-risk patients in Europe between 2020 and 2021: the multinational observational SANTORINI study. Lancet Reg Health Eur 29:100624 10.1016/j.lanepe.2023.100624 PMC10119631 37090089

[21] Nishino M, Egami Y, Sugino A, Kobayashi N, Abe M, Ohsuga M et al (2025) Factors correlated with non-early achievement of target low-density lipoprotein cholesterol level using PCSK9 inhibitors. Heart Vessels 40(12):1101–1108 10.1007/s00380-025-02571-1 40643625

[22] Marcusa DP, Giugliano RP, Park JG, de Lemos JA, Cannon CP, Sabatine MS (2021) Association of baseline low-density lipoprotein cholesterol and percentage low-density lipoprotein cholesterol reduction with statins, Ezetimibe, and PCSK9 Inhibition. JAMA Cardiol 6(5):582–586 10.1001/jamacardio.2020.6184 PMC7666429 33185670

[23] Mazdeyasnan D, Birs A, Chiou T, Diano L, Wilkinson MJ (2025) Insights from a real-world experience with inclisiran at a large United States lipid clinic. J Clin Lipidol 19(4):812–818 10.1016/j.jacl.2025.06.015 40670197

[24] Newman CB, Tobert JA (2023) Targeting PCSK9 with antibodies and gene silencing to reduce LDL cholesterol. J Clin Endocrinol Metab 108(4):784–790 10.1210/clinem/dgac708 36469793

[25] Corsini A, Ginsberg HN, Chapman MJ (2025) Therapeutic PCSK9 targeting: inside versus outside the hepatocyte? Pharmacol Ther 268:108812 10.1016/j.pharmthera.2025.108812 39947256

[26] Khattak S, Ochoa-Ferraro A, Khan N, George S, Khan SQ, Townend JN et al (2025) Relative efficacy of alirocumab, evolocumab, inclisiran, and bempedoic acid on lipids in patients with cardiovascular disease or familial hypercholesterolaemia. J Clin Med. 10.3390/jcm14227946 PMC12653900 41302982

