# Supplementary material for: Real-world lipid outcomes after switching PCSK9-targeting therapies in heterozygous familial hypercholesterolemia: the SHIFT-FH study
Source: Intern Emerg Med. 2026 Jun 13;21(7):2501–9. doi: 10.1007/s11739-026-04417-8 (PMC13694442; doi:10.1007/s11739-026-04417-8)
Supplement: Supplementary file 1 [file 11739_2026_4417_MOESM1_ESM.docx]

**Supplementary online material**

**Real-world lipid outcomes after switching PCSK9-targeting therapies in heterozygous familial hypercholesterolemia: the SHIFT-FH study**

Ilenia Lorenza Calcaterra^1,2*^, Carmine De Luca^1,2*^, Luigi Junior Valletta^1,2^, Guido D’Errico ^1,2^, Martina Ferrandino^3^, Giovanna Cardiero^3^, Maria Donata Di Taranto ^3^, Giuliana Fortunato ^3^, Gabriella Iannuzzo^1,2^, Antonella Tufano ^1,2^§, Matteo Di Minno^1,2^§

^1^Department of Clinical Medicine and Surgery, University of Naples Federico II, Naples, Italy; ^2^Regional reference Centre for treatment of Dyslipidaemia; ^3^Department of Molecular Medicine and Medical Biotechnologies, University of Naples Federico II, and CEINGE-Biotecnologie avanzate Franco Salvatore, Naples, Italy,

* These authors contributed equally to this work

§ These authors contributed equally to this work

**Table of contents**

| **Supplementary Figure 1** | **Patient flow diagram.** |
| --- | --- |
| **Supplementary Figure 2** | **Spaghetti plot of individual LDL-C trajectories across study time points (T0, T1, T2).** |
| **Supplementary Table 1** | **LDL-R mutation patterns distribution** |

**Supplementary Figure 1. Patient flow diagram.**

Of 228 patients with clinical suspicion of heterozygous familial hypercholesterolemia (HeFH), 155 met the Dutch Lipid Clinic Network score ≥6 and were screened for eligibility. A total of 107 were excluded (HbA1c >7%: n=18; secondary hyperlipidaemia: n=29; combined hyperlipidaemia: n=48; malignant neoplasia: n=12), resulting in 48 patients enrolled in the study.

**Supplementary Figure 2. Spaghetti plot of individual LDL-C trajectories across study time points (T0, T1, T2).**

Each line represents an individual patient. Blue lines indicate group 1 (G1) and orange lines indicate group 2 (G2). Bold lines represent group mean values at each time point.

**Supplementary Table 1. Frequency of *LDLR* mutations found in heterozygous FH patients.**

| **Nucleotide** | **Protein** | **Mutation type** | **Mutation classification** | **N** |
| --- | --- | --- | --- | --- |
| c.1135T>C | p.Cys379Arg | Missense | Defective | 7 |
| c.1646G>A | p.Gly549Asp | Missense | Defective | 5 |
| c.1775G>A | p.Gly592Glu | Missense | Defective | 5 |
| c.953G>T | p.Cys318Phe | Missense | Defective | 5 |
| c.1130G>T | p.Cys377Phe | Missense | Defective | 2 |
| c.1567G>A | p.Val523Met | Missense | Defective | 2 |
| c.2054C>T | p.Pro685Leu | Missense | Defective | 3 |
| c.1027G>A | p.Gly343Ser | Missense | Defective | 1 |
| c.1731G>C | p.Trp577Cys | Missense | Defective | 1 |
| c.1739C>T | p.Ser580Phe | Missense | Defective | 1 |
| c.352G>T | p.Asp118Tyr | Missense | Defective | 1 |
| c.1698_1704delinsGCCCAAT | p.Ile566_Leu568delinsMetProAsn | Small deletion without frameshift | Defective | 1 |
| c.2312-3C>A | p.Ala771_Ile796del | Splicing | Null | 10 |
| c.1586+1G>A | p.Thr454_Gly529del and p.Gly529_Phe530ins22 | Splicing | Null | 3 |
| c.313+5G>A | p.Leu64_Pro105delinsSer | Splicing | Null | 1 |

The table reports the nucleotide and protein changes, mutation type, functional classification, and number of carriers for each LDLR variant detected in the cohort. Variants were classified according to their predicted functional effect on LDL receptor activity as defective or null. The number of patients carrying each variant is reported in the last column (N).
